# Estimating the Seroincidence of Scrub Typhus using Antibody Dynamics after Infection

**DOI:** 10.4269/ajtmh.23-0475

**Published:** 2024-06-11

**Authors:** Kristen Aiemjoy, Nishan Katuwal, Krista Vaidya, Sony Shrestha, Melina Thapa, Peter Teunis, Isaac I. Bogoch, Paul Trowbridge, Stuart D. Blacksell, Daniel H. Paris, Tri Wangrangsimakul, George M. Varghese, Richard J. Maude, Dipesh Tamrakar, Jason R. Andrews

**Affiliations:** ^1^ Department of Public Health Sciences, University of California Davis School of Medicine, Davis, California;; ^2^ Department of Microbiology and Immunology, Faculty of Tropical Medicine, Mahidol University, Bangkok, Thailand;; ^3^ Dhulikhel Hospital, Kathmandu University Hospital, Dhulikhel, Nepal;; ^4^ Center for Global Safe Water, Sanitation and Hygiene, Hubert Department of Global Health, Rollins School of Public Health, Emory University, Atlanta, Georgia;; ^5^ Department of Medicine, University of Toronto, Toronto, Ontario, Canada;; ^6^ Michigan State University College of Human Medicine, Grand Rapids, Michigan;; ^7^ Mahidol-Oxford Tropical Medicine Research Unit, Faculty of Tropical Medicine, Mahidol University, Bangkok, Thailand;; ^8^ Centre for Tropical Medicine and Global Health, Nuffield Department of Medicine, University of Oxford, Oxford, United Kingdom;; ^9^ Department of Medicine, Swiss Tropical and Public Health Institute, Basel, Switzerland;; ^10^ Department of Clinical Research, University of Basel, Switzerland;; ^11^ Department of Infectious Diseases, Christian Medical College, Vellore, India;; ^12^ The Open University, Milton Keynes, United Kingdom;; ^13^ School of Public Health, Li Ka Shing Faculty of Medicine, University of Hong Kong, Hong Kong;; ^14^ Division of Infectious Diseases and Geographic Medicine, Stanford University School of Medicine, Stanford, California

## Abstract

Scrub typhus, a vector-borne bacterial infection, is an important but neglected disease globally. Accurately characterizing the burden is challenging because of nonspecific symptoms and limited diagnostics. Prior seroepidemiology studies have struggled to find consensus cutoffs that permit comparisons of estimates across contexts and time. In this study, we present a novel approach that does not require a cutoff and instead uses information about antibody kinetics after infection to estimate seroincidence. We use data from three cohorts of scrub typhus patients in Chiang Rai, Thailand, and Vellore, India, to characterize antibody kinetics after infection and two population serosurveys in the Kathmandu Valley, Nepal, and Tamil Nadu, India, to estimate seroincidence. The samples were tested for IgM and IgG responses to *Orientia tsutsugamushi*-derived recombinant 56-kDa antigen using commercial enzyme-linked immunosorbent assay kits. We used Bayesian hierarchical models to characterize antibody responses after scrub typhus infection and used the joint distributions of the peak antibody titers and decay rates to estimate population-level incidence rates in the cross-sectional serosurveys. Median responses persisted above an optical density (OD) of 1.8 for 23.6 months for IgG and an OD of 1 for 4.5 months for IgM. Among 18- to 29-year-olds, the seroincidence was 10 per 1,000 person-years (95% CI, 5–19) in Tamil Nadu, India, and 14 per 1,000 person-years (95% CI: 10–20) in the Kathmandu Valley, Nepal. When seroincidence was calculated with antibody decay ignored, the disease burden was underestimated by more than 50%. The approach can be deployed prospectively, coupled with existing serosurveys, or leverage banked samples to efficiently generate scrub typhus seroincidence estimates.

## INTRODUCTION

Scrub typhus, an acute febrile illness caused by the bacterium *Orientia tsutsugamushi*, is an important, underrecognized etiology of fever.^1^ Once thought to be restricted to the “tsutsugamushi triangle,” a region spanning from Russia to Pakistan, Australia, and Japan, recent studies have identified scrub typhus transmission in South America, Africa, and the Middle East.^2^^–^^4^ Infections occur when trombiculid mite chiggers (larvae) enter a host’s skin through hair follicles and feed on lysed skin tissue. The mites, both vectors and reservoirs of *O. tsutsugamushi*, feed on various mammals, including humans and rodents.^5^

In humans, symptoms are nonspecific and include fever, myalgia, headache, gastrointestinal symptoms, and rash. An eschar is a specific indicator of infection but is easily missed on clinical examination and may not always be present.^6^ Although infection can be effectively treated with antibiotics, when treatment is delayed, it can spread to organs and become severe, with case fatality rates reaching 12–13%.^7^^,^^8^

Indirect immunofluorescence assays (IFAs) have been the mainstay of scrub typhus diagnostics for decades.^9^ Standardized criteria for diagnosing scrub typhus involve either an acute IgM IFA titer of 1:3,200 or a 4-fold increase to at least 1:3,200 between acute- and convalescent-phase samples.^10^^,^^11^ The IFA method, requiring specialized equipment, skilled personnel, and both acute- and convalescent-phase samples, presents significant challenges, especially in low-resource settings, limiting its feasibility for routine diagnostics and surveillance. Enzyme-linked immunosorbent assays (ELISAs) have demonstrated high sensitivity and specificity and are more logistically feasible, positioning them as a more practical alternative to IFAs.^12^ InBios manufactures a commercially available IgM and IgG ELISA kit for scrub typhus that uses a recombinant p56-kDa type-specific antigen. When compared with IFA, the InBios IgM ELISA with a screening cutoff of 0.6 had a sensitivity of 84.2% and a specificity of 98.3% among patients presenting between 5 and 11 days after fever onset.^13^

Determining where and among whom scrub typhus transmission occurs is critical to inform public health interventions and research priorities. Clinical incidence underestimates the true underlying burden of disease because of nonspecific symptoms and the lack of affordable and accurate diagnostics.^14^ Periodic serosurveillance studies across countries of endemicity have demonstrated significant heterogeneity in seroprevalence within and between countries.^15^^,^^16^ However, directly comparing seroprevalences is not straightforward because of differences in the age distributions of each sampled population and the serologic assay as well as uncertainty in antibody-waning patterns.

Here, we apply a novel analytic approach to estimate scrub typhus seroincidence using antibody decay information from confirmed cases. The decay of antibody concentrations defines a timescale for inferring when an infection occurred. This approach does not depend on classification using cutoffs, which have been difficult to derive for scrub typhus across locations with varying forces of infection.^17^ We first model longitudinal IgG and IgM antibody responses to the *O. tsutsugamushi* 56-kDa antigen among confirmed scrub typhus cases in Thailand and India and then use these parameters to estimate scrub typhus seroincidence from cross-sectional population serosurveys in Nepal and India.

## MATERIALS AND METHODS

### Study populations and enrollment.

We used data from three cohorts of scrub typhus patients in Chiang Rai, Thailand, and Vellore, India, to characterize antibody kinetics after infection and two population serosurveys in the Kathmandu Valley, Nepal, and Tamil Nadu, India, to estimate seroincidence (Supplemental Table 1).

#### Scrub typhus cases.

We used antibody responses measured from confirmed scrub typhus patients enrolled in three studies: two in Thailand and one in India. The first study in Thailand included 35 children less than 18 years old who were hospitalized with fever (temperature higher than 37.5°C) or a history of fever within the last 14 days at Chiang Rai Prachanukroh Hospital.^18^ Enrollment occurred between July 2015 and August 2016. Confirmed scrub typhus infections were defined by meeting at least one of the following criteria: 1) a positive polymerase chain reaction (PCR) or culture result from either a blood or eschar sample, 2) a 4-fold rise in the IgM titer to ≥1:3,200 in paired serum or plasma samples, or 3) a single IgM titer of ≥1:3,200 in an acute-phase serum or plasma sample. Eleven of the 35 children had a systemic complication, and one child with multiorgan failure died. Venous blood samples were collected at baseline and at 2, 12, and 52 weeks after enrollment, centrifuged, and serum was stored at −80°C and transported to Bangkok for diagnostic processing.

The second study in Thailand included 43 hospitalized patients >15 years old from a fever surveillance study also at the Chiang Rai Prachanukhao Hospital.^14^^,^^19^ Enrollment occurred from August 2007 to August 2008. Patients confirmed to be infected with scrub typhus were defined by meeting at least one of the following criteria: 1) in vitro isolation of *O. tsutsugamushi*, 2) a ≥4-fold rise in IgM titer in paired serum samples when tested by the IFA, or 3) a positive result in at least two of the three PCR assays described by Paris et al.^19^ Data on clinical severity were not available for this cohort. Venous blood samples were collected at admission and stored at −80°C until testing. Blood samples were collected at baseline only.

In India, venous blood samples were collected from individuals diagnosed with scrub typhus who were >18 years old and who sought care at Christian Medical College (CMC) Teaching Hospital in Vellore, India, between December 2011 and March 2015.^20^ Confirmed patients with scrub typhus were defined by a positive IgM ELISA (optical density [OD], >0.8) and/or a positive PCR for *O. tsutsugamushi*. Data on clinical severity were not available for this cohort. The blood samples were collected from retrospective cases with diagnoses made between 2 months and 3.5 years earlier. Venous blood samples were collected in the patient’s household, transported to the laboratory on ice, and centrifuged, and aliquoted serum was stored at −80°C and processed within 2 weeks of collection.

#### Population samples.

In Nepal, a geographically random, population-based cross-sectional sample of individuals aged 0 to 25 years was enrolled from the catchment areas of Kathmandu Medical College and Teaching Hospital in Kathmandu (urban) and of Dhulikhel Hospital in Kavrepalanchok, Nepal (periurban and rural).^21^ Within catchment areas, geographically defined grid clusters were randomly selected and all households in each cluster were enumerated and then individuals were randomly selected. If the selected individual was not available or did not consent, a consenting person in that same age stratum was selected. Only one participant was selected per household. We collected capillary blood samples from consenting participants onto TropBio filter papers (Cellabs Pty Ltd., Brookvale, New South Wales, Australia). The samples were air-dried for at least 12 hours at room temperature and then stored with desiccant in individual plastic bags at −20°C until processing. Study participants were enrolled between February 2019 and January 2021. In India, a cross-sectional serosurvey for scrub typhus conducted from September 2014 to December 2014,^22^ enrolling adults ≥18 years of age in the Vellore District of Tamil Nadu. A two-stage clustered sample design was used to randomly select communities and individuals while ensuring that 60% of the sample individuals were from rural communities to reflect the broader population demographics of Tamil Nadu.^22^ Only one participant was selected per household. Venous blood was collected from consenting participants in the household, transported on ice, and centrifuged and serum was stored at −80°C until tested. A timeline of enrollment for both population studies is shown in *Supplemental Figure 1*.

#### Laboratory methods.

All samples were tested using IgM and IgG responses to *O. tsutsugamushi*-derived recombinant 56-kDa antigen using the Scrub Typhus Detect ELISA kit (InBios International, Inc., Seattle, WA)^14^ and performed according to the manufacturer’s instructions. All samples were tested at a 1:100 dilution, and the results were read at 450 nm using a microplate reader (Thermo Scientific Multiskan FC) to generate a final OD result (OD at 450 nm). In Nepal, two filter paper protrusions were submerged in 133 *µ*L of 1× phosphate-buffered saline (PBS)–0.05% Tween buffer overnight at 4°C, then centrifugation at 10,000 × *g*. The resulting supernatant was recovered and considered to be equivalent to a 1:10 dilution of serum.

## STATISTICAL ANALYSES

We estimated seroincidence in two ways: 1) by deriving it from the age-dependent seroprevalence, and 2) as a function of the longitudinal antibody dynamics after infection.

### Method 1.

Because no consensus cutoffs are established for the InBios Scrub Detect ELISA, we used finite mixture models to determine the IgG seropositivity cutoffs using the mean plus 2 SD of the lower distribution.^23^ We calculated IgG seroprevalence as the proportion of individuals classified as IgG seropositive. We then used an exponential survival model to derive seroincidence as the rate required to achieve a given seroprevalence at a given age [Equations (1) and (2)], where π(a) equals the seroprevalence at age (a) and λ equals the seroincidence rate.

$$\pi \left(a\right)=1-e^{-\lambda a}$$ (1)

$$\lambda =\frac{\left(-\log_{10}\;(1-\pi \left(a)\right)\right)}{a}$$ (2)

We fit a generalized linear model to the binomial seropositivity outcome conditional on age with a complementary log-log link and estimated seroincidence from the model’s intercept term.^24^^,^^25^ This approach assumes that antibody responses do not wane after exposure and that seroincidence is constant over time. To evaluate how seropositivity changes over age, we fit generalized additive models^26^ with a cubic spline for age and simultaneous CIs using a parametric bootstrap of the variance-covariance matrix of the fitted model parameters.^27^

### Method 2.

We used a method established by Teunis et al.^28^^–^^30^ to estimate seroincidence using information about antibody decay from confirmed cases. After exposure to a pathogen, the adaptive immune system responds by rapidly producing antibodies to inactivate and remove pathogens. When the infection has been cleared (pathogens removed), antibody production is downregulated and serum antibody levels slowly return to baseline levels. Given this specific seroresponse pattern, serum antibody levels may be used as indicators of the time passed since exposure. By analyzing serum antibody concentrations in a population sample, we can infer the incidence rate, i.e., the number of new infections per unit time, in that population. Specifically, we used a two-phase within-host model to characterize the seroresponse post-scrub typhus infection as an exponential rise in antibody levels followed by power function decay, starting with rapidly declining antibody levels followed by a long period of very slow drift towards baseline^28^^,^^29^^,^^31^ We used a Bayesian hierarchical framework to fit the above models for anti-*O. tsutsugamushi* 56-kDa IgG and IgM^28^^,^^29^ and generated joint distributions using Markov chain Monte Carlo sampling, allowing for individual variation. The model prior distributions are listed in Supplemental Table 2. We implemented the models in JAGS v. 4.3.2 called from R v. 4.1.3.^32^

The above seroresponse model can be used to predict antibody levels in a cross-sectional population sample where the distribution of antibody levels depends on the infection rate. High infection rates correspond to short time periods between infections, so that a random subject tends to be sampled a short time after infection, with little time for antibody decay. Assuming that incident infections occur as a Poisson process with rate lambda (λ), the expected distribution of antibody levels in a cross-sectional population sample can be calculated.^29^^,^^30^^,^^33^ Using this distribution, a likelihood function can be defined for the observed population sample. We generated maximum likelihood estimates and 95% CIs for the incidence rate (λ) using IgM and IgA separately and jointly by combining their likelihood functions. We considered two sources of noise in the observed serologic responses: measurement noise and biologic noise as detailed by Teunis and van Eijkeren.^30^ Measurement noise refers to the variation in observed antibody levels when samples are tested in duplicate across different plates. We did not have replicates available for these data and instead used an estimate for measurement noise of 20%. Biological noise denotes the variation in background levels of antibodies, e.g., nonspecific antibodies reacting with the assay antigen. For our study, we used biological noise parameters of 0.74 for IgG and 0.32 for IgM, derived from the 75%ile of the discrete mixture analysis approximating the seronegative component.

To investigate the sensitivity of our incidence estimates to longitudinal case data, we modeled the longitudinal data separately for India and Thailand, compared the distributions of modeled kinetic parameters (peak, decay rate, and decay shape), and estimated the seroincidence for the Tamil Nadu, India, population using Tamil Nadu, India-specific kinetic parameters.

### Reproducibility.

Code to reproduce the seroincidence estimates are available as an article within the serocalculator R package.^34^

## RESULTS

Antibody responses were measured from 280 patients with confirmed scrub typhus infections: 77 in Chiang Rai, Thailand, and 203 in Vellore, India.^20^ In the pediatric cohort in Chiang Rai, Thailand, four samples were collected from each participant between 0 and 400 days after symptom onset. In the Vellore, India, cohort, 195 participants had one sample collected and 8 participants had two samples collected, all of which were collected between 72 and 1,300 days after presentation. In the adult cohort in Thailand, just one sample was collected between 1 and 11 days after symptom onset (median, 5.5 days) and only IgM responses were measured. The median age of all participants was 43 years (interquartile range [IQR], 26–55; range, 1.2–84): 47 years in India (IQR, 35–57), 43 years in the adult cohort in Thailand (IQR, 35–51), and 6.6 years (IQR, 4–10) in the pediatric cohort in Thailand (Table 1).

**Table 1:** Summary of longitudinal scrub typhus patient data

Parameters	Tamil Nadu, India: Adults (*n* = 203)	Chiang Rai, Thailand: Pediatric Patients (*n* = 35)	Chiang Rai, Thailand: Adults (*n* = 42)	Overall (*N* = 280)
Age, years				
Median (IQR)	47 (35–57)	6.6 (4.0–10)	43 (35–51)	43 (26–55)
Min/Max	19–79	1.2–13	15–84	1.2–84
Missing	0 (0%)	0 (0%)	1 (2.4%)	1 (0.4%)
Days from symptom onset to first visit				
Median (IQR)	530 (370–890)	10 (7.5–12)	5.5 (4.0–7.0)	390 (12–770)
Min/Max	72–1,300	3.0–19	0.15–11	0.15–1,300
Days from symptom onset to last visit				
Median (IQR)	230 (220–250)	350 (350–370)	–	350 (340–360)
Min/Max	170–310	10–400	–	10–400
Missing	195 (96.1%)	0 (0%)	–	237 (84.6%)
Number of visits				
Median (IQR)	1.0 (1.0–1.0)	4.0 (4.0–4.0)	1.0 (1.0–1.0)	1.0 (1.0–1.0)
Min/Max	1.0–2.0	1.0–4.0	1.0–1.0	1.0–4.0

IQR = interquartile range; min/max = minimum/maximum.

**Figure 1.**
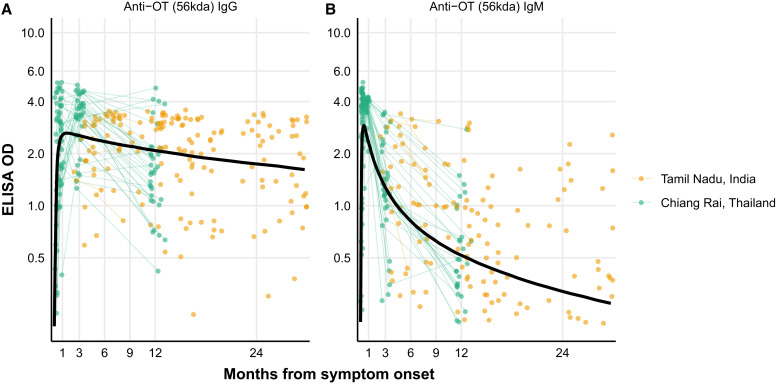
Kinetics of IgG (**A**) and IgM (**B**) responses among scrub typhus cases. Longitudinal antibody dynamics were modeled from enzyme-linked immunosorbent assay (ELISA)-measured antibody responses using Bayesian hierarchical models. The points are the observed individual antibody concentrations; each point indicates one sample, and samples from a patient with more than one sample are connected with lines. The dark solid line indicates the median for the model-fitted antibody decay concentrations. OD = optical density; OT = *Orientia tsutsugamushi*.

Among cases, median IgG responses were elevated above the mixture model cutoff for 23.6 months after symptom onset (Figure 1A). An IgM OD of 1 has been suggested as a reasonable cutoff to indicate a recent infection.^35^^,^^36^ In our study population, median IgM responses were elevated above an OD of 1 for 4.45 months after infection, 25% of the population had responses of >1 for 10.5 months, and 10% had elevated responses of >1 for 22.3 months (Figure 1B). IgM responses decayed at a rate of OD 1.89 (95% credible interval [CrI], 0.87–4.26) per month compared with 0.11 (95% CrI, 0.04–0.29) per month for IgG. Modeled IgM responses peaked at 6.5 days (95% CrI, 3.6–11.7) after symptom onset. IgG responses peaked at OD 11.99 (95% CrI, 6.6–22.05) 11 days (95% CrI, 5.7–20.6) after symptom onset (Supplemental Table 3). The residuals for the within-host two-phase model are displayed in Supplemental Figure 3.

**Figure 2.**
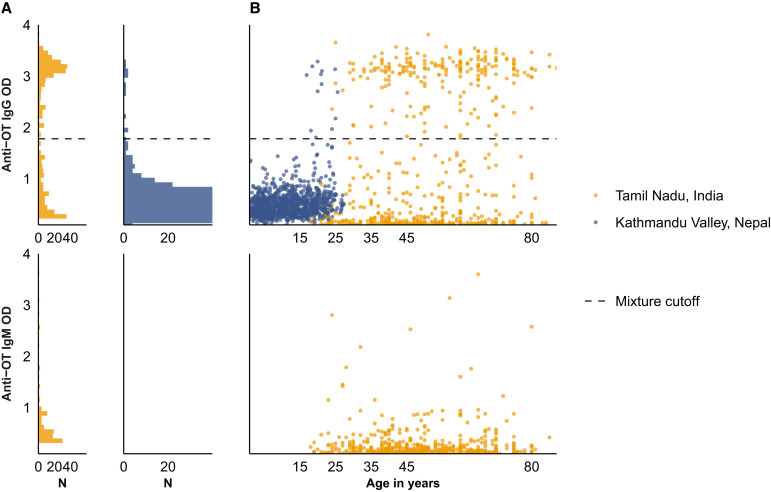
Quantitative antibody responses among population serosurveys in Nepal and India. Anti-*Orientia tsutsugamushi* (OT) IgM and IgG responses are measured using enzyme-linked immunosorbent assays and depicted on the *y* axis of all plots. The horizontal dashed line denotes the mixture model cutoff. (**A**) Distribution of responses among the population-based samples in India and Nepal. (**B**) Antibody responses among the population-based samples in India and Nepal as a function of age. OD = optical density.

For the population data, antibody responses were measured from 721 participants in Tamil Nadu, India, and 1,105 participants in Kathmandu and Kavre Districts, Nepal (Figure 2). The median age of participants was 49 years in India (IQR, 40–62) and 11 in Nepal (IQR, 5.5–17). In India, 63.2% (456/721) of the participants were female, compared with 48.7% (538/1,105) in Nepal (Table 2). Figure 2 depicts the distribution of quantitative antibody responses (Figure 2A) and the change of antibody responses with age (Figure 2B) in each site.

**Table 2:** Population data summary

Parameters	Tamil Nadu, India (*N* = 721)	Kathmandu Valley, Nepal (*N* = 1,105)
Age, in years		
Median (IQR)	49 (40–62)	11 (5.5–17)
Min/Max	18–87	0.90–27
Age, in years, categorical		
0–17	0 (0%)	876 (79.3%)
18–29	59 (8.2%)	229 (20.7%)
30–49	302 (41.9%)	0 (0%)
50–89	360 (49.9%)	0 (0%)
Sex		
Female	456 (63.2%)	538 (48.7%)
Male	265 (36.8%)	567 (51.3%)
Occupation		
Agriculture	85 (11.8%)	0 (0%)
Labor and manufacturing	181 (25.1%)	14 (1.3%)
Minor, ≤18 years	1 (0.1%)	880 (79.6%)
None	343 (47.6%)	152 (13.8%)
Other	21 (2.9%)	21 (1.9%)
Professional	44 (6.1%)	26 (2.4%)
Service	46 (6.4%)	12 (1.1%)

IQR = interquartile range; Min/Max = minimum/maximum.

**Figure 3.**
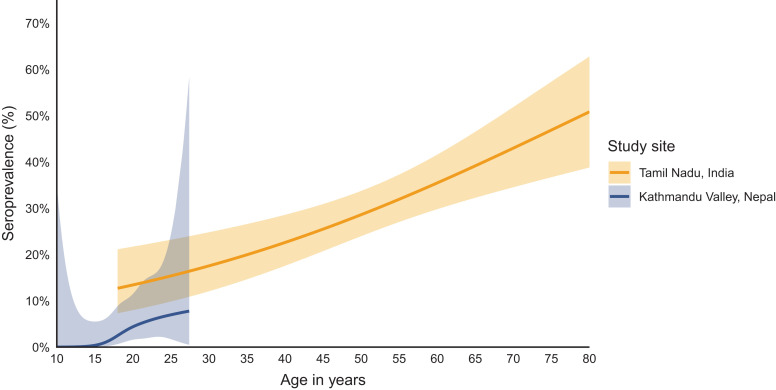
Age-dependent IgG seroprevalence. Seroprevalence is depicted as a function of age. Seroprevalence was determined using the mixture-model-derived cutoff for IgG responses. The age-dependent seroprevalence was modeled using a generalized additive model with a cubic spline for age and simultaneous CIs using a parametric bootstrap of the variance-covariance matrix of the fitted model parameters.

In both India and Nepal, the seroprevalence of scrub typhus infection increased with age (Figure 3). In Tamil Nadu, India, the overall seroprevalence was 29.8% (95% CI, 26.5–33.2%), increasing from 10.2% (95% CI, 2.4–17.9%) among 18- to 29-year-olds to 38.1% (95% CI, 33.0–43.1%) among 50- to 89-year-olds. In Kathmandu and Kavre, Nepal, the overall seroprevalence was 1.2% (95% CI, 0.5–1.8%), increasing from 0.1% (95% CI, 0–0.3%) among 0- to 17-year-olds to 5.2% (95% CI, 2.3–8.1%) among 18- to 29-year-olds (Table 3).

**Table 3:** Age-specific seroprevalence and seroincidence in the Kathmandu Valley, Nepal, and Tamil Nadu, India, population-based serosurveys*

Location and Age Group (Years)	*N*	*n* IgG Seropositive	% IgG Seroprevalence (95% CI)	Seroincidence per 1,000 Person-years (95% CI)		
				IgG Antibody Kinetics	IgG and IgM Antibody Kinetics	Derivative of Age-Dependent IgG Seroprevalence
Tamil Nadu, India						
0–17	0	0	–	–	–	–
18–29	59	6	10.2% (2.4–17.9)	10 (5–19)	14 (8–22)	4 (2–10)
30–49	302	72	23.8% (19.0–28.7)	15 (12–19)	14 (11–17)	7 (5–9)
50–89	360	137	38.1% (33.0–43.1)	29 (24–35)	19 (16–23)	8 (6–9)
Overall	721	215	29.8% (26.5–33.2)	20 (18–23)	16 (14–18)	7 (6–8)
Kathmandu Valley, Nepal						
0–17	876	1	0.1% (0–0.3)	6 (4–9)	–	0 (0–1)
18–29	229	12	5.2% (2.3–8.1)	14 (10–20)	–	3 (1–4)
30–49	0	0	–	–	–	–
50–89	0	0	–	–	–	–
Overall	1,105	13	1.2% (0.5–1.8)	8 (7–11)	–	1 (1–2)

* * n* () IgG seropositive = number of individuals in the population sample who were seropositive using the IgG mixture model cutoff of 1.8. IgG seroprevalence = the percentage of individuals who were seropositive divided by the total population in each stratum. Seroincidence using IgG antibody kinetics = the seroincidence rate per 1,000 person-years using longitudinal IgG antibody dynamics (method of Teunis and van Eijkeren^30^). Seroincidence using IgG and IgM antibody kinetics = the seroincidence rate per 1,000 person-years using longitudinal IgG and IgM antibody dynamics (method of Teunis and van Eijkeren^30^). Seroincidence using age-dependent IgG seroprevalence = seroincidence derived from the age-dependent cumulative hazard of IgG seroprevalence.

In Tamil Nadu, India, the overall scrub typhus seroincidence rate among 18- to 87-year-olds was 20 per 1,000 person-years (95% CI, 18–23) using IgG responses. Seroincidence rose with age, increasing from 10 (95% CI, 5–19) per 1,000 person-years among 18- to 29-year-olds to 29 (95% CI, 24–35) among 50- to 89-year-olds. In the Kathmandu Valley of Nepal, the overall seroincidence among 0- to 27-year-olds was 8 (95% CI, 7–11) per 1,000 person-years, also rising with age from 6 (95% CI, 4–9) per 1,000 person-years among 0- to 17-year-olds to 14 (95% CI, 10–20) among 18- to 29-year-olds. In both Nepal and India, the seroincidence among 18- to 29-year-olds using antibody dynamics was more than double the seroincidence derived from the age-dependent IgG seroprevalence, 2.4 times higher in India and 4.7 times higher in Nepal (Table 3). IgM responses were available only from the study in India; there, the seroincidence using IgM was similar to that using IgG, and the CIs using IgM and IgG together were more narrow than those using IgG alone (Table 3).

**Figure 4.**
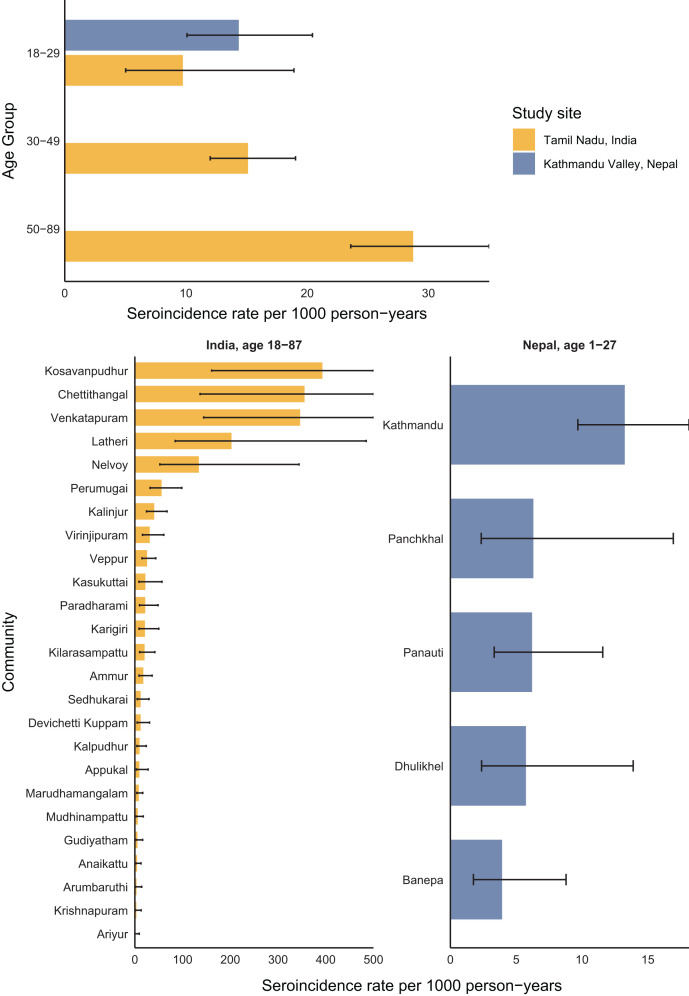
Seroincidence by age and community. Seroincidence rates per 1,000 person-years are shown across age strata and communities in a comparison of the India and Nepal population samples. Seroincidence is estimated using IgG responses based on modeled antibody kinetics. Bars represent incidence estimates per 1,000 person-years, and lines indicate the 95% CIs.

At both sites, scrub typhus seroincidence varied by community. In Tamil Nadu, India, community-level seroincidence ranged from 0 to 392 per 1,000 person-years and was higher than 300 per 1,000 person-years in three communities. In the Kathmandu Valley in Nepal, seroincidence was highest in Kathmandu (Figure 4). There were no significant differences in seroincidence across sex (Supplemental Figure 2).

In a sensitivity analysis comparing model antibody dynamics in Chiang Rai, Thailand, and Vellore, India, there was substantial overlap in the distributions of peak antibody response, decay rate, and decay shape (Supplemental Figure 3). The incidence estimates for Tamil Nadu, when fit with only the CMC, Vellore, cohort data, were similar to those fit with the combined cohort data (Supplemental Figure 4).

## DISCUSSION

In this study, we have extended the use of an established analytic approach to a novel context to estimate the seroincidence of scrub typhus using antibody decay information from confirmed cases and cross-sectional serosurveys. Seroincidence, the number of new infections in a population per year, characterizes transmission intensity and informs where and among whom the infection burden is highest. We found a high seroincidence of scrub typhus in both study populations that increased with age, underscoring the importance of addressing the burden of scrub typhus infection in these regions. We characterized the persistence of antibodies after infection and using this information to interpret population-level responses. Our results demonstrate that employing a cutoff method and disregarding antibody decay underestimates the true burden of scrub typhus infection.

Most cross-sectional seroepidemiologic studies of scrub typhus rely on seroprevalence as a marker of infection, employing a cutoff threshold to dichotomize IgG responses and calculating the proportion of the population with values above the threshold.^9^^,^^16^^,^^37^ Antibody responses in a population at any given time will be a function of exposure, the age of the population, and antibody decay shape and rate. For example, in our study populations, the overall seroprevalence was much higher in India than in Nepal; however, in India, the median age of patients was 49 years while the median age was 11 years in Nepal. Despite differences in overall seroprevalence rates, the discrepancies in age-specific seroprevalence rates between the two populations were less marked. This underlines the importance of considering age-specific seroprevalence rates when comparing seroprevalences of different populations, as the age distribution can have a considerable impact on the observed seroprevalence. Our novel analytical approach, which leverages information about antibody decay rates to generate seroincidence estimates, provides a more accurate representation of scrub typhus infection in these regions, highlighting the need for targeted public health interventions and research.

In both India and Nepal, seroprevalence increased with age, a finding that has been observed in other scrub typhus serosurveys.^22^^,^^38^ An increase in seroprevalence with age can result from a gradual accumulation in IgG responses over time or from a genuinely elevated risk of infection in older age groups. Both arguments have been put forth for scrub typhus seroepidemiology, with the suggestion that elevated risk in older ages is attributable to changes in skin health, immune system function, and physiological processes.^35^ We argue that reporting seroincidence rather than seroprevalence helps to disentangling the effects of IgG accumulation over age versus age-specific risk. Incorporating antibody dynamics into the calculation of seroincidence, as demonstrated in this paper, further elucidates age-specific changes in infection risk by accounting for decaying antibody levels. In our study populations, we observed an increase in seroincidence with age, suggesting genuine increased risk of infection among older ages. This finding highlights the importance of using seroincidence to better understand the age-specific risk of infection and to inform targeted public health interventions.

Defining antibody dynamics and persistence is crucial for interpreting population-level seroresponses to *O. tsutsugamushi*. In our study, we found that median IgG responses remained elevated above a mixture-model cutoff of 1.7 OD for nearly 2 years. These findings align with a recent study by Schmidt et al.,^35^ which reported that half of the cases had persistent IgG antibodies beyond 2 years. Interestingly, IgM responses persisted longer than anticipated, with half of the participants having an OD of >1 for 4.5 months after symptom onset and 10% of participants having an OD of >1 for 22.3 months. This extended persistence contrasts with the findings of Schmidt et al., where half of the participants had an IgM OD of >1 after 2.7 months and 10% had an OD of >1 after 7.7 months. A potential explanation for this discrepancy could be the differences in modeling approaches. Our power function model allows for the shape of the decay to be nonexponential, permitting a slower decay at longer times since infection. In contrast, a restricted cubic spline approach requires a constant rate and shape of decay. The persistence of IgM revealed in our study suggests that it would be a good complement to IgG to identify recent infections and improve the precision around seroincidence estimates. Indeed, for the India data where IgM results were available, the seroincidence estimates using both IgM and IgG were consistent, yielding similar estimates for the seroincidence, and had narrower confidence intervals.

One of the key strengths of our approach lies in its ability to account for the inherent heterogeneity of antibody responses, as well as measurement and biologic noise. Traditional seroepidemiological methods, which rely on a single cutoff value for seroprevalence and seroincidence, fail to incorporate these sources of variability. By explicitly integrating the heterogeneity in antibody responses, our method captures the distribution of responses among individuals across ages, clinical severity, and levels of immune function within our sampled cohorts. Additionally, our approach takes into account the measurement noise associated with the ELISA and the biologic noise that stems from nonspecific antibody binding. This more comprehensive incorporation of uncertainty leads to more accurate and robust estimates of scrub typhus seroincidence, which in turn better informs public health interventions and research priorities.

Our findings have several implications for public health. First, we reveal a substantial burden of scrub typhus beyond what is captured by the reporting of clinical cases. In Tamil Nadu, India, a recently conducted population-based fever surveillance study reported a clinical incidence of 0.8 cases per 1,000 person-years, defined as the incidence of scrub typhus among febrile individuals who sought care or were hospitalized.^38^ By applying our seroincidence estimates derived from IgG and IgM antibody dynamics, we estimate that there are around 14 subclinical infections for every clinical case in the population surrounding CMC Hospital, not accounting for health care-seeking behavior. This inflation factor is less than that estimated by Devamani et al.,^36^ who reported a seroincidence of 44 per 1,000 person-years. This study was conducted in a population that was purposefully selected for its elevated numbers of clinical scrub typhus cases, which could explain the differences.^36^ Population-based clinical incidence estimates for scrub typhus are not currently available for the study population in Nepal seroincidence studies like ours are useful for identifying areas with a substantial scrub typhus burden, thereby informing the allocation of resources for enhanced clinical diagnostic capacity and targeted surveillance efforts. Furthermore, our study challenges the notion that scrub typhus is solely a rural disease. We observed elevated incidence rates in both urban and periurban communities in Nepal and India. However, we could not ascertain whether exposure occurred at the place of residence or was linked to rural exposure, highlighting the need for further research targeting these populations and investigating exposures. Expanding our understanding of the distribution and risk factors associated with scrub typhus in various settings will help inform the development of more effective and targeted prevention and control measures, ultimately reducing the burden of disease in affected populations. For example, these results can be used to identify several communities with extremely high scrub typhus seroincidence rates, which could be prioritized for prevention and awareness education campaigns.

There are several limitations to our study that warrant consideration. First, the population serosurveys in India and Nepal were conducted over different time periods, with the Indian survey spanning a few months and the Nepalese survey taking place over a year. This discrepancy could introduce variability in the observed seroprevalence and seroincidence rates between the two populations due to seasonal differences in scrub transmission. Second, factors such as age, geography, clinical severity, immunological status, and coinfection could lead to variations in antibody decay patterns that could alter seroincidence estimates. We did not have data available on coinfections and immunological status and thus could not investigate these variables. Clinical severity data were available only for the Thai pediatric cohort. In our sensitivity analysis, antibody decay parameters did not differ significantly between the Thailand and India cohorts, which also represented embedded differences in age. Third, the antibody decay rate and peak antibody levels are likely influenced by subsequent infections. In areas with a high force of infection, where individuals are frequently reexposed, antibody responses might exhibit slower decay. This could lead to an overestimation of the seroincidence rate. Given the constraints of our longitudinal case data, with between one and four follow-up visits, our ability to detect reexposures was limited. However, the wide age spectrum within our dataset suggests a possibility that certain individuals might have experienced prior infections before being included. This implies that the reported decay rate potentially represents a blend of initial and subsequent infections, influencing the seroincidence rate derived from it. To fully understand these dynamics, future prospective studies with extended longitudinal follow-up visits will help ascertain the frequency of reexposures and to delineate their antibody response patterns. Fourth, the Nepal population study collected capillary blood stored on filter paper. For other antibodies like those to salmonella typhi and paratyphi responses are similar when serum (venous blood) and dried blood spots (capillary blood) are compared,^21^^,^^39^ but as far as we know, this has not been rigorously assessed for antibody responses to the 56kda antigen. It is possible that antibody responses in filter paper/capillary blood could be attenuated relative to those in serum, which would underestimate the burden in Nepal. Fifth, our cohorts did not include individuals with mild or subclinical infections, and therefore we may not have captured the full spectrum of antibody responses. A study enrolling individuals across a broader spectrum of health settings would paint a more comprehensive picture of scrub typhus infection dynamics. Finally, while the studies in Chiang Rai, Thailand, adhered to a specific scrub typhus infection criteria,^11^^,^^19^ the ELISA cutoff of 0.8 used for the CMC, Vellore, cases, may not offer the same level of specificity. Prolonged IgM responses after infection, as documented in this study and others, raise concerns about potential misdiagnosis in febrile patients with elevated IgM levels, possibly originating from a previous infection. In this scenario, we may have included cases later in their course of infection, which could underestimate IgG and IgM peak and decay rates in the samples from India. Future prospective studies that follow the same infection criteria across multiple sites would be valuable compare antibody dynamics across populations. Despite these limitations, our study offers a new seroepidemiological tool to characterize scrub typhus burden and highlights the importance of accounting for antibody dynamics and heterogeneity.

Scrub typhus remains an important but underrecognized etiology of acute fever with endemicity expanding globally. There is a critical need for low-cost, accurate tools to quantify the burden of scrub typhus infections to inform public health decision-making. We describe a serosurveillance approach that can efficiently generate population-level scrub typhus seroincidence estimates from cross-sectional serosurveys. This methodology offers a promising new tool for informing targeted prevention and control strategies, ultimately contributing to a more effective response to scrub typhus in endemic regions worldwide.

## Supplemental Materials

10.4269/ajtmh.23-0475 Supplemental Materials
