# Supplementary material for: Estimating the Seroincidence of Scrub Typhus using Antibody Dynamics after Infection
Source: Am J Trop Med Hyg. 2024 Jun 11;111(2):267–76. doi: 10.4269/ajtmh.23-0475 (PMC11310618; doi:10.4269/ajtmh.23-0475)
Supplement: Supplemental Materials [file tpmd230475.SD1.pdf]

SUPPLEMENTAL MATERIAL

Estimating the seroincidence of scrub typhus using antibody dynamics following infection

Authors: Kristen Aiemjoy, Nishan Katuwal, Krista Vaidya, Sony Shrestha, Melina Thapa, Peter Teunis, Isaac I. Bogoch, Paul Trowbridge, Stuart D. Blacksell, Daniel Paris, Tri Wangrangsimakul, George M Varghese, Richard Maude, Dipesh Tamrakar, Jason R. Andrews

Corresponding Author:
Kristen Aiemjoy
Assistant Professor
Division of Epidemiology
Department of Public Health Sciences,
University of California Davis School of Medicine, Davis, CA, USA
Department of Microbiology and Immunology, Faculty of Tropical Medicine, Mahidol University, Bangkok Thailand
kaiemjoy@ucdavis.edu

SUPPLEMENTAL FIGURE 1: SAMPLING COLLECTION OVER TIME AND GEOGRAPHIC UNIT
Each point indicates an individual who was sampled at a given time (X-axis) and their quantitative anti-OT56kda IgG antibody response (Y-axis). The color of the dot represents the participant's age in years.

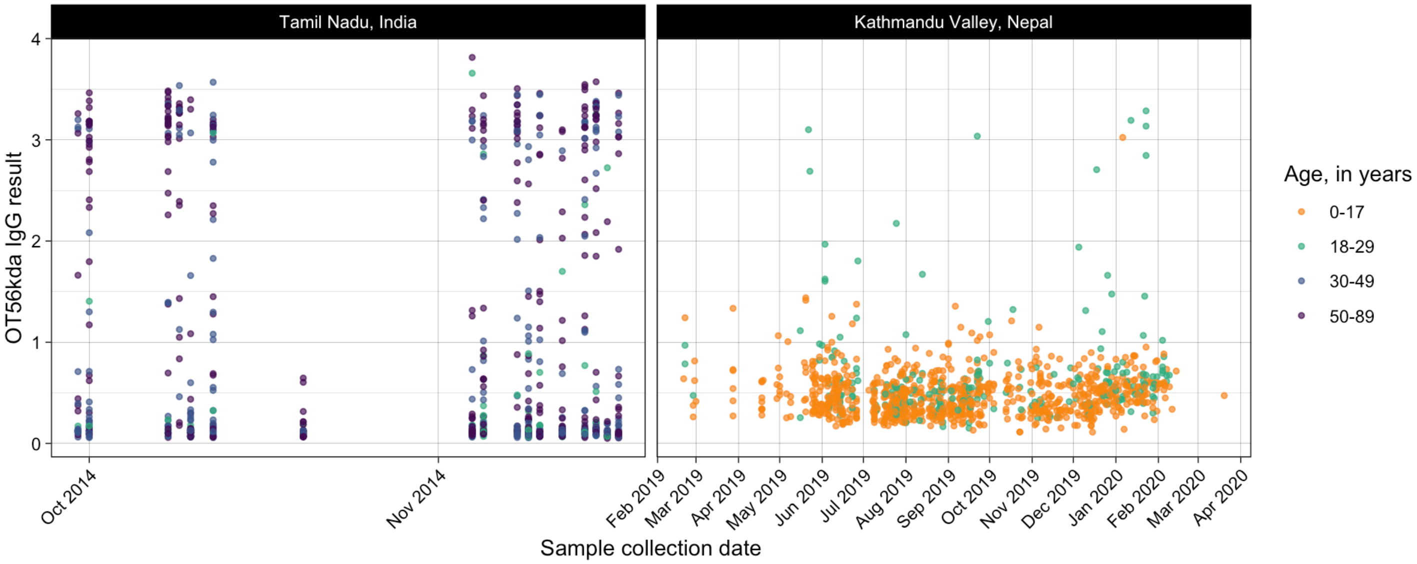

SUPPLEMENTAL TABLE 1: DATA SOURCES

Study	Data Source
Nepal population data	**Study design described here:** Aiemjoy et al., 2022. Estimating typhoid incidence from community-based serosurveys: a multicohort study. *The Lancet Microbe 3*: e578–e587<br><br>***Scrub typhus serology reported in this paper for the first time**
Thailand pediatric case data	**Study design described here:** Wangrangsimakul et al., 2020. Clinical Characteristics and Outcome of Children Hospitalized With Scrub Typhus in an Area of Endemicity. *Journal of the Pediatric Infectious Diseases Society 9*: 202–209<br><br>***Longitudinal seroresponse data reported for the first time here**
Thailand adult case data	**Data previously published here:** Blacksell et al., 2015. Diagnostic Accuracy of the InBios Scrub Typhus Detect Enzyme-Linked Immunoassay for the Detection of IgM Antibodies in Northern Thailand. *Clin Vaccine Immunol 23*: 148–154
India longitudinal data	**Data previously published here:** Varghese et al., 2018. Kinetics of IgM and IgG antibodies after scrub typhus infection and the clinical implications. *International Journal of Infectious Diseases 71*: 53–55
India population data	**Data previously published here:** Trowbridge et al., 2017. Prevalence and risk factors for scrub typhus in South India. *Tropical Medicine & International Health 22*: 576–582

SUPPLEMENTAL TABLE 2: PRIOR DISTRIBUTIONS FOR MODEL PARAMETERS BY IMMUNOGLOBULIN TYPE (IGM AND IGG)

Hyperprior and Prior Specifications for Bayesian Model

Parameter	Hyperprior Mean	Hyperprior Precision	Prior Variance
		**IgM**	
log(y0)	-2	5.0	10
log(y1)	1	0.5	10
log(t1)	1	5.0	10
log(alpha)	-1	1.0	50
log(shape)-1	-3	0.5	10
		**IgG**	
log(y0)	-2	5.0	10
log(y1)	1	0.5	10
log(t1)	1	5.0	10
log(alpha)	-1	1.0	50
log(shape)-1	-3	0.5	10

SUPPLEMENTAL TABLE 3: MODELED ANTIBODY KINETIC PARAMETERS

	Median (IQR)	
**Parameter**	**IgG**	**IgM**
Decay rate (per year)	0.11 (0.04-0.29)	1.89 (0.87-4.26)
Decay shape	1.30 (1.14-1.59)	2.19 (1.80-2.73)
Time to peak (days)	11.99 (6.62-22.05)	6.50 (3.66-11.70)
Baseline response	0.13 (0.07-0.23)	0.06 (0.03-0.10)
Peak response	2.81 (2.08-3.72)	4.87 (3.41-6.90)

SUPPLEMENTAL FIGURE 2: SCRUB TYPHUS SEROINCIDENCE RATES BY GENDER AND STUDY SITE

Points indicate the seroincidence rate for Orientia Tstusugamushi per 100,000 person years by sex, age strata and study site. The lines indicate the 95% confidence intervals. Seroincidence was estimated using quantitative ant-OT56kda IgG antibody responses among representative population-based serosurveys in Tamil Nadu, India and the Kathmandu Valley, Nepal.

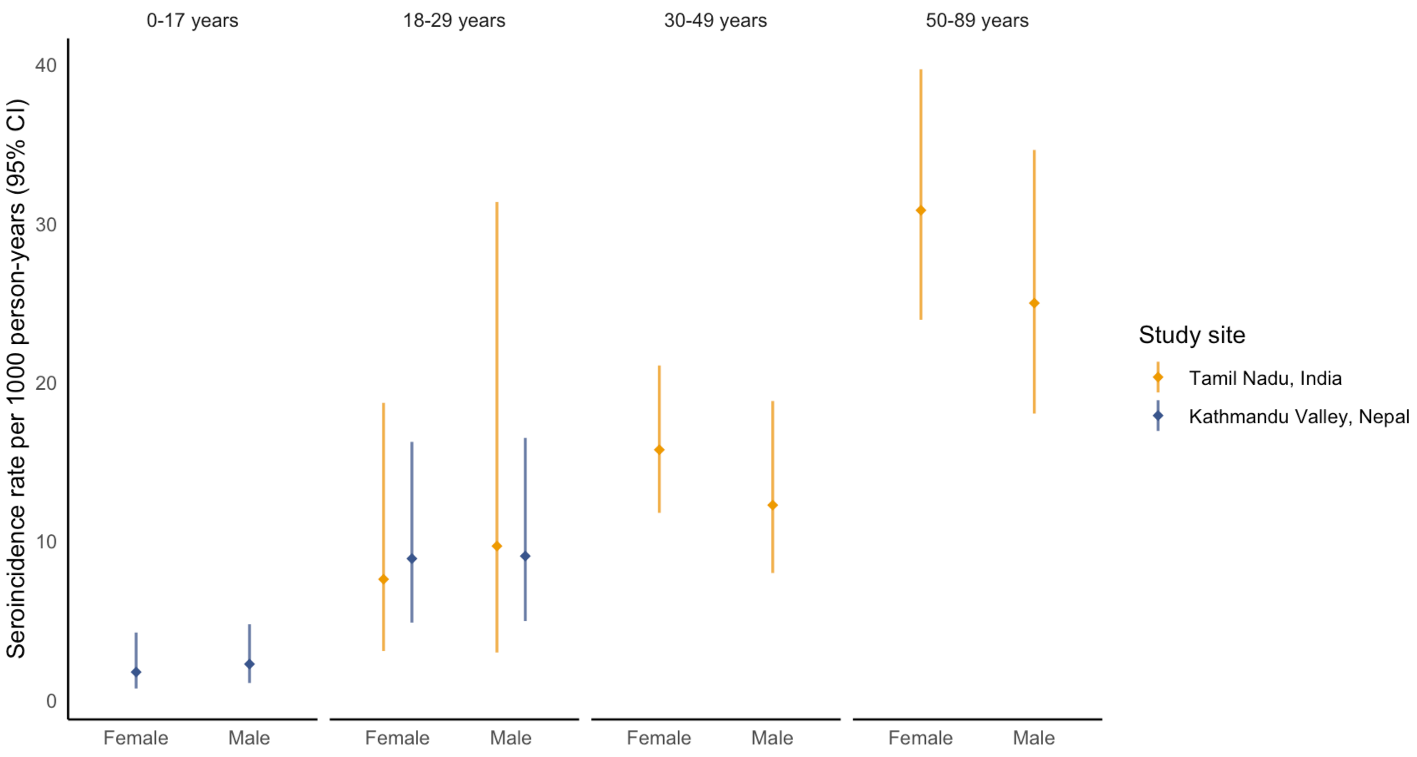

SUPPLEMENTAL FIGURE 3: COMPARISON OF LONGITUDINAL ANTIBODY KINETIC PARAMETERS BETWEEN STUDY COUNTRIES

Distribution of modeled OT56kda IgG and IgM antibody decay kinetics comparing confirmed scrub typhus cases enrolled from Chiang Rai, Thailand, and Tamil Nadu, India.

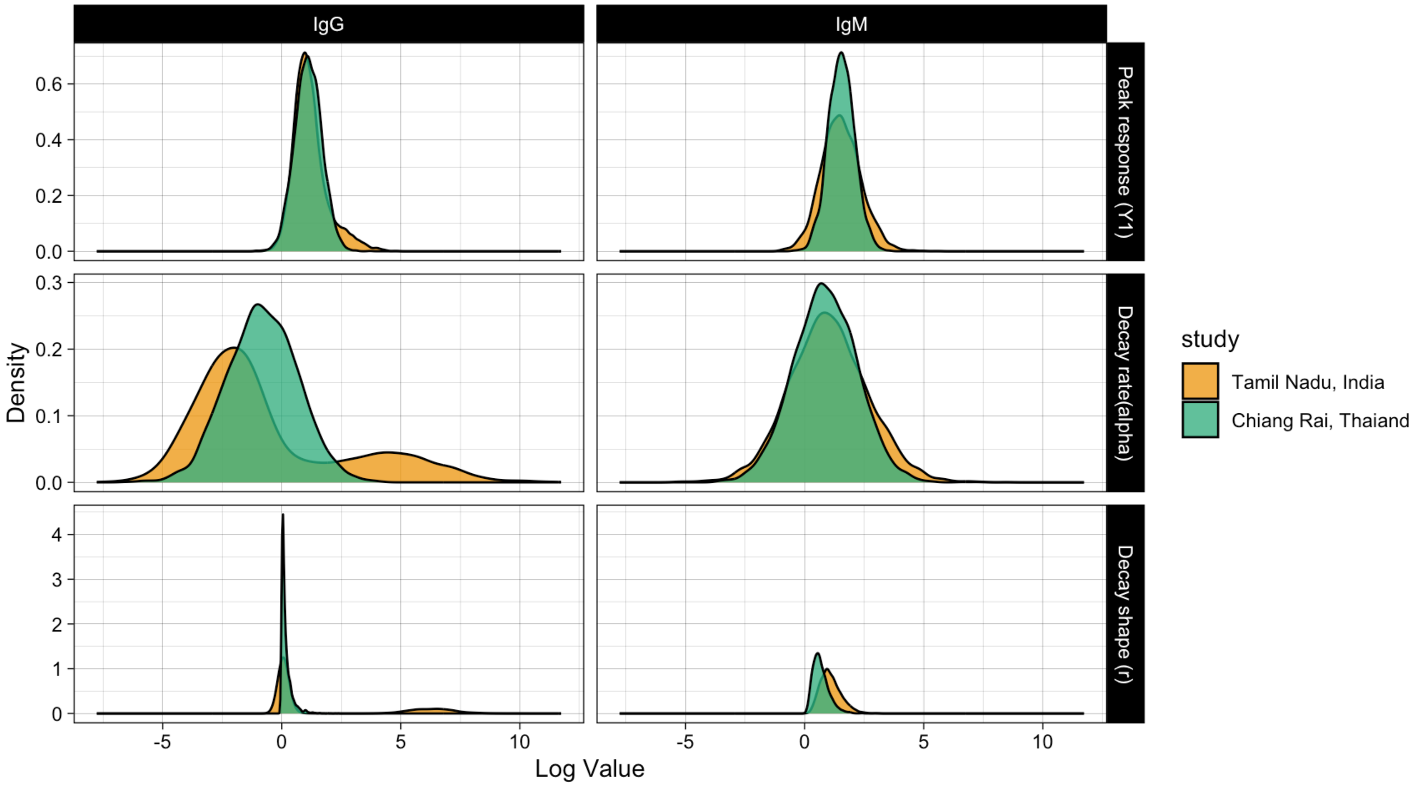

SUPPLEMENTAL FIGURE 4: ORIENTIA TSUTSUGAMUSHI SEROINCIDENCE RATES FOR THE TAMIL NADU POPULATION SEROSURVEY COMPARING TWO LONGITUDINAL ANTIBODY KINETICS MODELS

Points indicate the Orientia Tstusugamushi seroincidence rate per 100,000 person-ears, and the lines indicate the 95% confidence intervals. Seroincidence was estimated using quantitative ant-OT56kda IgG antibody responses among representative population-based serosurveys and antibody kinetics modeled from confirmed scrub typhus cases. The solid lines use antibody kinetics modeled from confirmed cases in Tamil Nadu, India alone, and the dashed lines used antibody kinetics from Tamil Nadu, India, and Chiang Rai, Thailand.

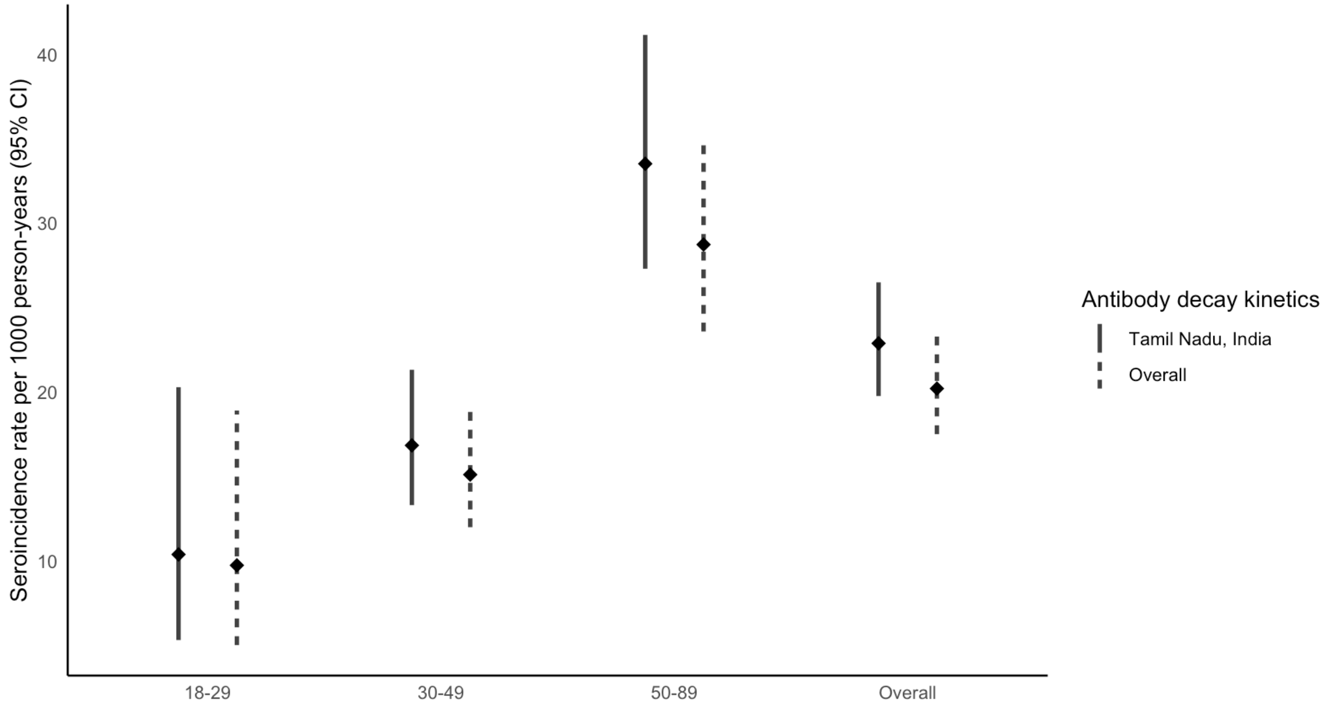

SUPPLEMENTAL FIGURE 5: RESIDUALS FROM BAYESIAN HIERARCHICAL MODEL OF ANTIBODY KINETICS

Residual plots for the fit of the modeled antibody kinetics to the observed longitudinal antibody responses. The y-axis indicates the log residual difference between the observed data and the fitted decay model.

OT56kda IgG	OT56kda IgG
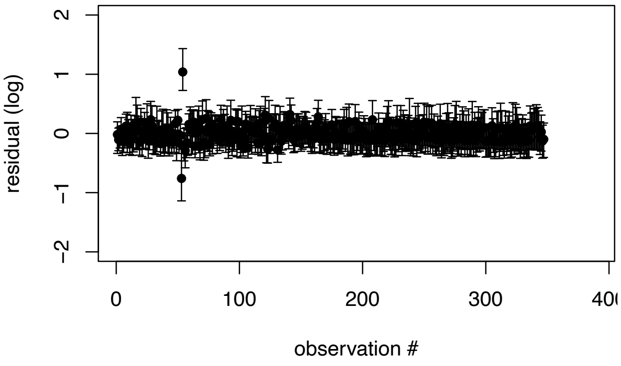	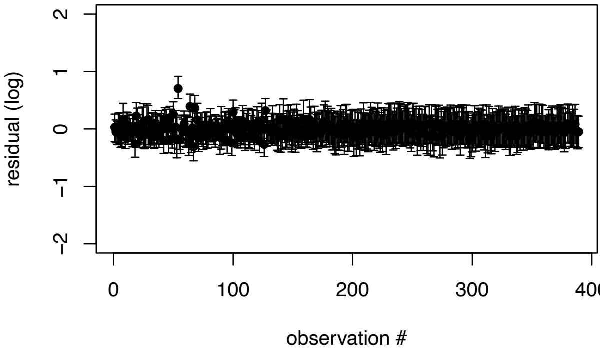
